# CD14-Dependent Monocyte Isolation Enhances Phagocytosis of *Listeria monocytogenes* by Proinflammatory, GM-CSF-Derived Macrophages

**DOI:** 10.1371/journal.pone.0066898

**Published:** 2013-06-11

**Authors:** Caroline Neu, Anne Sedlag, Carina Bayer, Sabine Förster, Peter Crauwels, Jan-Hendrik Niess, Ger van Zandbergen, Giada Frascaroli, Christian U. Riedel

**Affiliations:** 1 Institute of Microbiology and Biotechnology, University of Ulm, Ulm, Germany; 2 Institute of Virology, University Medical Center Ulm, Ulm, Germany; 3 Division of Immunology, Paul-Ehrlich-Institute, Federal Institute for Vaccines and Biomedicines, Langen, Germany; 4 Department of Visceral Medicine and Surgery, Inselspital, Bern, Switzerland; Duke University Medical Center, United States of America

## Abstract

Macrophages are an important line of defence against invading pathogens. Human macrophages derived by different methods were tested for their suitability as models to investigate *Listeria monocytogenes (Lm)* infection and compared to macrophage-like THP-1 cells. Human primary monocytes were isolated by either positive or negative immunomagnetic selection and differentiated in the presence of granulocyte macrophage colony-stimulating factor (GM-CSF) or macrophage colony-stimulating factor (M-CSF) into pro- or anti-inflammatory macrophages, respectively. Regardless of the isolation method, GM-CSF-derived macrophages (GM-Mφ) stained positive for CD206 and M-CSF-derived macrophages (M-Mφ) for CD163. THP-1 cells did not express CD206 or CD163 following incubation with PMA, M- or GM-CSF alone or in combination. Upon infection with *Lm*, all primary macrophages showed good survival at high multiplicities of infection whereas viability of THP-1 was severely reduced even at lower bacterial numbers. M-Mφ generally showed high phagocytosis of *Lm*. Strikingly, phagocytosis of Lm by GM-Mφ was markedly influenced by the method used for isolation of monocytes. GM-Mφ derived from negatively isolated monocytes showed low phagocytosis of *Lm* whereas GM-Mφ generated from positively selected monocytes displayed high phagocytosis of *Lm*. Moreover, incubation with CD14 antibody was sufficient to enhance phagocytosis of *Lm* by GM-Mφ generated from negatively isolated monocytes. By contrast, non-specific phagocytosis of latex beads by GM-Mφ was not influenced by treatment with CD14 antibody. Furthermore, phagocytosis of *Lactococcus lactis*, *Escherichia coli*, human cytomegalovirus and the protozoan parasite *Leishmania major* by GM-Mφ was not enhanced upon treatment with CD14 antibody indicating that this effect is specific for *Lm*. Based on these observations, we propose macrophages derived by *ex vivo* differentiation of negatively selected human primary monocytes as the most suitable model to study *Lm* infection of macrophages.

## Introduction

*Listeria monocytogenes* (*Lm*) is a food-borne Gram-positive obligate intracellular pathogen that is able to cope with a wide range of environmental conditions and is thus found in various of habitats [1]. In humans, the disease caused by *Lm* is termed listeriosis and manifests primarily in immunocompromised individuals, pregnant women, new-borns, and elderly patients with a mortality of 20–30% in these at risk groups [2]. Infections with *Lm* are usually acquired upon consumption of contaminated food products and thus the first habitat inside the host is the gastrointestinal tract [3]. *Lm* is able to cross the intestinal barrier, subsequently enters the blood and lymph stream, and finally colonizes liver and spleen where it is primarily phagocytosed by resident macrophages [4]. *Lm* either actively enters host cells using a number of proteins of the internalin family or is taken up passively by phagocytosis. Following uptake, *Lm* is able to disrupt the vacuolar membrane by the secretion of two phospholipases, PlcA and PlcB, and the pore-forming toxin listeriolysin O (LLO) [5]. This results in the release of *Lm* into the cytoplasm where it starts to replicate and spread from one cell to another by hijacking the host cell actin cytoskeleton [6].

Macrophages play a central role in activating and finely balancing the pro- and anti-inflammatory pathways of the host immune system to mount effective host responses against invading pathogens. *In vivo*, macrophage differentiation is driven by GM- and M-CSF [7], [8]. High levels of GM-CSF induce a pro-inflammatory phenotype resulting in high IL–12 secretion. These pro-inflammatory cells are also termed M1 macrophages. By contrast, M-CSF polarizes macrophages to an anti-inflammatory phenotype characterized by IL–10 secretion, which is referred to as M2 macrophages [9]. Under normal conditions tissue macrophages show an anti-inflammatory phenotype, mostly devoted to tissue repair [8]. Additionally, blood monocytes are likely predisposed towards an anti-inflammatory phenotype due to high M-CSF levels in plasma [10], [11]. During infections, GM-CSF levels are increased stimulating the generation and release of monocyte/macrophage precursors in the bone marrow and skewing the macrophage population towards a pro-inflammatory phenotype [8], [12].

Pro- and anti-inflammatory macrophage populations can be differentiated *ex vivo* from human blood monocytes stimulated by either GM-CSF or M-CSF [13]–[16]. GM-CSF monocyte-derived macrophages (GM-Mφ) or M-CSF monocyte-derived macrophages (M-Mφ) have distinct morphology, pathogen susceptibility, and effector functions [8], [9], [15]–[17], which phenotypically correspond to M1 and M2 macrophages, respectively. Both states of differentiation are reversible [16], [18], [19].

In the gastrointestinal mucosa, resident macrophages display a predominantly anti-inflammatory phenotype presumably to prevent permanent and excessive stimulation of the immune system as a result of constant exposure to commensal and pathogenic microbes [20]–[22]. During acute infection most microbes drive macrophages towards a pro-inflammatory phenotype, whereas chronic disease mostly propagates the switch from a pro- to an anti-inflammatory state of differentiation [23].

So far, most studies on interactions between macrophages and *Lm* are performed in murine models. Due to a number of differences between the immune response in mice and humans, especially with respect to macrophages [12], [24]–[26] there are limitations in directly translating the results obtained with murine models to the human system.

Thus, studies in primary human cells might be a more appropriate approach to investigate human innate immune responses to infection with *Lm* but also other pathogens. Accordingly, in the present study we evaluate different models for human macrophages to study *Lm* infection.

## Materials and Methods

### Ethics statement

Human cells used in this study were isolated from buffy coats of anonymous healthy blood donors and buffy coats were purchased from the Institute of Clinical Transfusion Medicine, University of Ulm. The Institutional Review Board of the University of Ulm approved experiments and informed written consent approving and authorizing the use of their material for research purposes was obtained from all donors.

### Cultivation of bacteria and eukaryotic cells

*Listeria monocytogenes* EDGe (*Lm*), *Escherichia coli* BL21 and *Lactococcus lactis* NZ9000 were grown at 37°C in brain heart infusion (BHI; Oxoid, Germany) medium. THP-1 cells were purchased from the American Type Culture Collection (ATCC® Number: TIB-202) and routinely cultured at 37°C in a 5% CO_2_ atmosphere in RPMI 1640 medium (Gibco Life Technologies, Germany) containing 10% fetal bovine serum (FBS, Sigma-Aldrich, Germany), 10 mM L-glutamine (PAA Laboratories, Germany), 1% (v/v) non-essential amino acid solution (NEAA; PAA Laboratories, Germany) with medium changed three times a week. THP-1 cells were passaged as recommended by the supplier. To generate macrophage-like cells, THP-1 cells were seeded at a density of 2×10^5^ cells cm^−2^ in 24 well tissue culture plates (BD Biosciences, Germany) and stimulated with 200 nM phorbol 12-myristate 13-acetate (PMA; Sigma-Aldrich, Germany) for 72 h prior to experiments.

### Generation of GM-Mφ and M-Mφ

Human monocytes were isolated from fresh buffy coats. Lymphocytes were prepared by density gradient centrifugation using lymphocyte separation medium (LSM 1077; PAA Laboratories, Germany). Monocytes were isolated from lymphocytes either by positive selection using magnetic CD14 MicroBeads (human; Cat# 130-050-201, Miltenyi Biotech, Germany) or by negative selection using Monocyte Isolation Kit II (human; Cat# 130-091-153, Miltenyi Biotech, Germany) according to the manufacturer's instructions. Where indicated, negatively selected monocytes were additionally incubated with CD14 microbeads or CD14 antibody (Cat# 130-091-242, Miltenyi Biotech, Germany) for 15 min at 4°C prior to further differentiation.

Following isolation, monocytes were cultured in Lumox® dishes (Sarstedt, Germany) for 7 days with RPMI 1640 medium containing 10% FBS, 10 mM L-glutamine, 1% (v/v) penicillin/streptomycin (PAA Laboratories, Germany) and 1% (v/v) NEAA. For differentiation, 50 ng ml^−1^ recombinant M-CSF (R&D Systems, Germany) or GM-CSF (Leukine®, Genzyme Corporation, UK) were added to the dishes. After 3 days medium was changed and growth factors were freshly added. On day 7 cells were detached by rinsing with phosphate-buffered saline (PBS; PAA Laboratories, Germany) and seeded at 2×10^5^ cells cm^−2^ into 24 well plates (BD Biosciences, Germany) unless stated otherwise. Prior to experiments, cells were grown over night in antibiotic-free medium containing growth factors. To minimize donor-dependent variations, in all experiments both GM-Mφ and M-Mφ were generated from the same donor. All experiments were performed in triplicates with cells from the same donor and at least three donors were tested.

### Flow cytometry

For fluorescence-activated cell sorting (FACS), cells were washed once with washing buffer (3% (v/v) FBS and 0.1% (w/v) NaN_3_ in PBS), resuspended in blocking buffer (3% (v/v) FBS; 5% (v/v) normal human AB serum, Cat# C11-020; PAA Laboratories, Germany and 0.1% NaN_3_ (w/v) in PBS) and incubated for 15 min at 4°C. 2×10^5^ cells were incubated with the following antibodies at appropriate dilutions for 30 min at room temperature (RT) in the dark: mouse anti-human CD206-PE (Cat# 555954), CD163-PE (Cat# 556018), CD14-FITC (Cat# 555397; all antibodies from BD Biosciences, Germany). IgG2a-FITC and IgG1-PE antibodies (Cat# 340394; BD Biosciences, Germany) were used as isotype controls. Subsequently, cells were washed, fixed in paraformaldehyde (PFA; 4% (v/v) in PBS; Sigma-Aldrich, Germany) and stored at 4°C in washing buffer until further use. FACS was performed in a FACSCalibur flow cytometer (BD Biosciences, Germany). In a typical experiment at least 10,000 events were recorded and data was analysed using BD CellQuest™ Pro software.

### Viability assay

Viability of the cells during experiments was monitored by classical 3-(4,5-Dimethylthiazol-2-yl)-2,5-diphenyltetrazolium bromide (MTT; Sigma-Aldrich, Germany) assay. Cells were incubated with 1 mg ml^−1^ (THP-1 cells) or 5 mg ml^−1^ (primary macrophages) MTT solution in PBS for 4 h at 37°C and then lysed in dimethyl sulfoxide (DMSO; Sigma-Aldrich). Absorbance of the dye was measured at 570 nm with a reference filter at 620 nm in an Infinite® M200 (Tecan, Germany) microtiter plate reader. Viability of uninfected cells was set to 100%.

### Gentamicin protection assay

Phagocytosis of bacteria by macrophages was measured by a gentamicin protection assay. Overnight cultures of either *Lm, E. coli* or *L. lactis* in BHI medium were diluted 1∶10 in fresh BHI and cultured to mid-exponential growth phase. Bacteria were washed once with PBS and adjusted to 1×10^9^ colony-forming units (CFU) per ml in RPMI medium. Macrophages were infected with bacteria at the indicated multiplicity of infection (MOI) in RPMI 1640 medium containing 10% FBS. To ensure comparable sedimentation and contact with macrophages, bacteria were centrifuged onto cells at 300×g for 1 min and phagocytosis was allowed by incubation for 1 h at 37°C under cell culture conditions (5% CO_2_). Then, supernatants were removed, fresh medium containing 10 µg ml^−1^ gentamicin (Gibco Life Technologies, Germany) was added and cells were incubated for 1 h at 37°C (5% CO_2_) to kill non-phagocytosed extracellular bacteria. Finally, cells were washed three times with 1× PBS (primary macrophages) or culture medium (THP-1 cells). For lysis, 1 ml 0.1% (v/v) Triton-X100 (Sigma-Aldrich, Germany) in H_2_O was added to each well. After incubation for 5 min, lysates were serially 10-fold diluted and 10 µl spots were platted in triplicates on BHI-Agar plates for enumeration. CFU were counted after incubation for 16 h at 37°C.

### Human cytomegalovirus (HCMV) infection assay

M- or GM-Mφ were seeded into 96 well plates (0.8×10^5^ cells per well) and infected with the endotheliotropic HCMV strain TB40E (kindly provided by C. Sinzger, University of Ulm, Germany) at an MOI of 5 infective particles per cell. TB40E virus stocks were produced by infected human foreskin fibroblasts (HFF) cultivated in minimal essential medium (MEM) with 10% FBS, 2 mM L-glutamine, 100 U ml^−1^ penicillin and 100 U ml^−1^ streptomycin as previously described [14]. 24 h post infection, cells were fixed with PFA (4% (v/v) in PBS) and permeabilized with 0.2% (v/v) Triton-X100. The immediate-early viral proteins IE 1-2 were detected with monoclonal antibodies (MAb E13; Argene-Biosoft, Verniolle, France) followed by incubation with an Alexa 488-conjugated goat anti-mouse secondary antibody (ICN Biomedical, Germany). Nuclei were counterstained with 4′,6′-diamino-2-phenylindole (DAPI; Roche Applied Science, Germany). Samples were imaged using an AxioObserver.Z1 (Carl Zeiss MicroImaging, Germany) and analysed using AxioVision software (version 4.6.1). HCMV infection rates were calculated by counting and correlating DAPI and IE 1-2 positive nuclei in five randomly selected microscopic fields for each experiment.

### Leishmania major infection assay

To generate green fluorescent parasites, *Leishmania major* (MHOM/IL/81/FEBNI) promastigotes were transfected with linearized pSSUint-eGFP (kindly provided by Dr. Toni Aebischer, Robert Koch Institute, Berlin, Germany) by electroporation as described for *Leishmania mexicana* [27]. Transgenic *L. major* 18Srrna::eGFP were cultured in RPMI 1640 medium supplemented with 5% FBS, 100 µg ml^−1^ penicillin/streptomycin, 2 mM glutamine, 10 mM HEPES (all supplements Biochrom, Germany), 50 µM beta-mercaptoethanol (Sigma Aldrich, Germany) and 20 µg/ml hygromycin B (InvivoGen, France) at 27°C in a humidified atmosphere containing 5% CO_2_.

For infection assays, promastigotes of fluorescent *L. major* parasites were harvested in the stationary growth phase. Prior to infection, parasite viability was assessed by Annexin V staining (Cat# A13202, Invitrogen Life Technologies, Germany) and flow cytometry. Infection of GM-Mφ and M-Mφ were performed as described [28]. Briefly, Mφ were co-incubated with *L. major* 18Srrna::eGFP promastigotes at an MOI of 10 parasites per cell for 3 h. Subsequently, extracellular *Leishmania* parasites were removed by centrifugation (10 min, 140×g, 20°C) and infection rates were determined 18 h post infection by FACS analysis.

### Non-specific phagocytosis of latex beads

Phagocytosis of latex beads was performed essentially as described elsewhere [29]. In brief, carboxylate-modified red fluorescence latex beads with a mean diameter of 2 µm (Cat# L3030; Sigma-Aldrich, Germany) were opsonized in RPMI 1640 including 10% human serum for 30 min at 37°C. Macrophages were incubated with opsonized latex beads at a bead∶cell ratio of 10∶1 for 2 h at 37°C and 5% CO_2_. Cells were then detached using Alfazyme (PAA Laboratories, Germany), washed twice with PBS containing 3% (v/v) FBS and 0.1% (w/v) NaN_3_ and then analysed by flow cytometry.

### Immunofluorescence assay

Infected macrophages were analysed for intracellular bacteria by fluorescence microscopy. For this purpose infections were performed in μ-slides (ibidi, Germany). Following three washing steps after the gentamicin treatment cells were fixed in 4% (v/v) PFA for 20 min at RT and stained with 300 nM DAPI in PBS for 5 min. Cells were permeabilized with 0.5% saponin (AppliChem, Germany) in PBS for 10 min at RT and intracellular *Lm* bacteria were stained with a rabbit anti-Listeria polyclonal antibody (Cat# ABIN122781, antibodies-online, Germany) followed by incubation with Alexa555-coupled secondary goat anti-rabbit antibody (Cat# A21428, Invitrogen Life Technologies, Germany). Stained samples were imaged using an AxioObserver.Z1 and analysed using AxioVision software. For quantification of intracellular bacteria at least 150 cells were counted per sample.

### Statistics

For each experiment monocytes were isolated from at least three independent donors by positive and negative selection in parallel and used to generate M- and GM-Mφ. Macrophages of each donor were analysed in triplicate and data was analysed by pair wise comparisons using Student's *t*-test as indicated in the figure legends with *p* values <0.05 considered significant.

## Results

### Surface marker expression of macrophages

**Figure 1:**
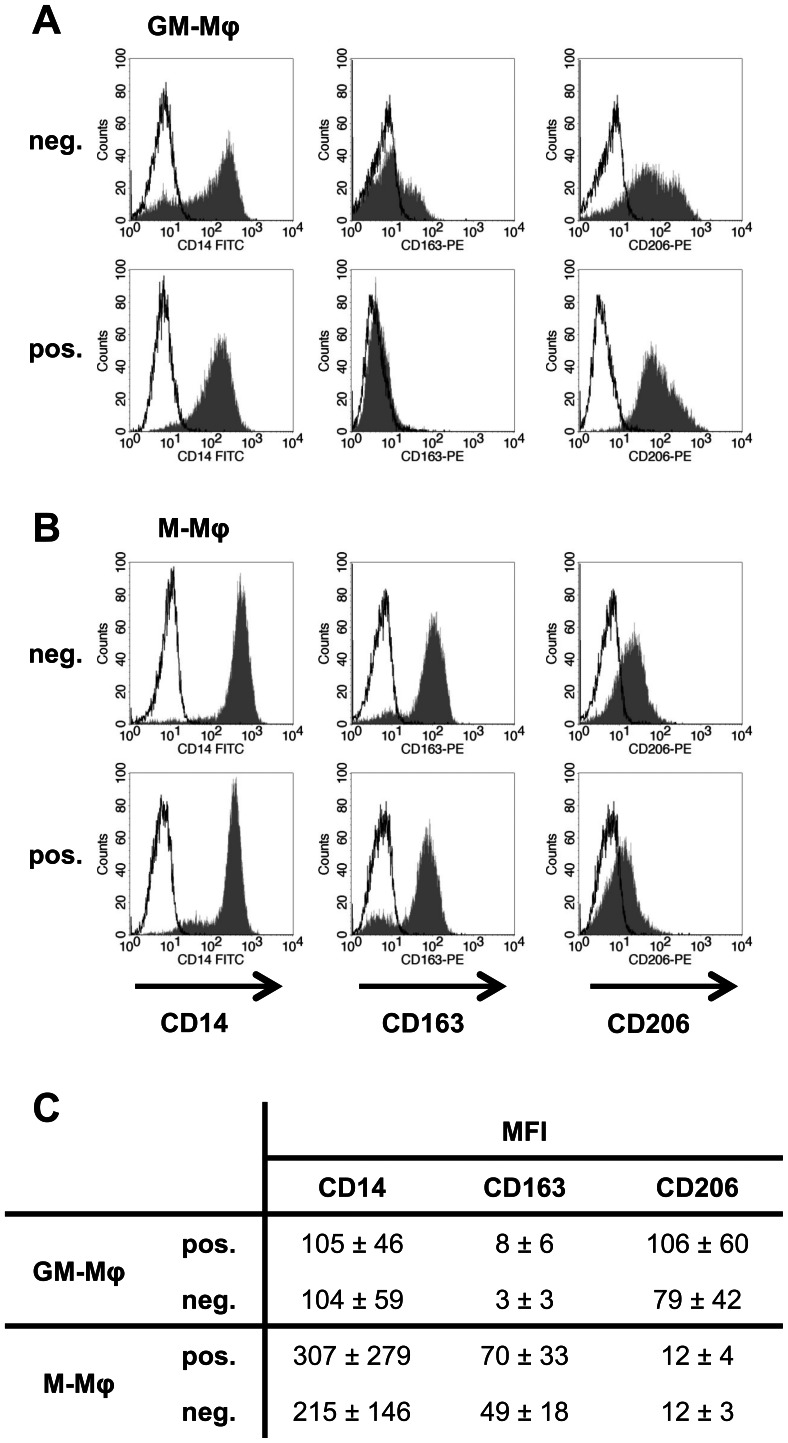
Surface expression of CD14, CD163 and CD206 on GM-Mφ (A) and M-Mφ (B) derived from monocytes isolated by negative (neg.) or positive (pos.) selection. Histogram plots show surface marker staining as hatched areas and isotype controls as black lines. Results from one representative donor are shown and similar results were obtained for each isolation and phenotype with cells of at least three donors. (C) Fluorescence intensity of surface marker staining on primary macrophages. Values are mean fluorescence intensity (MFI) ± standard deviation obtained with cells of three different donors.

**Figure 2:**
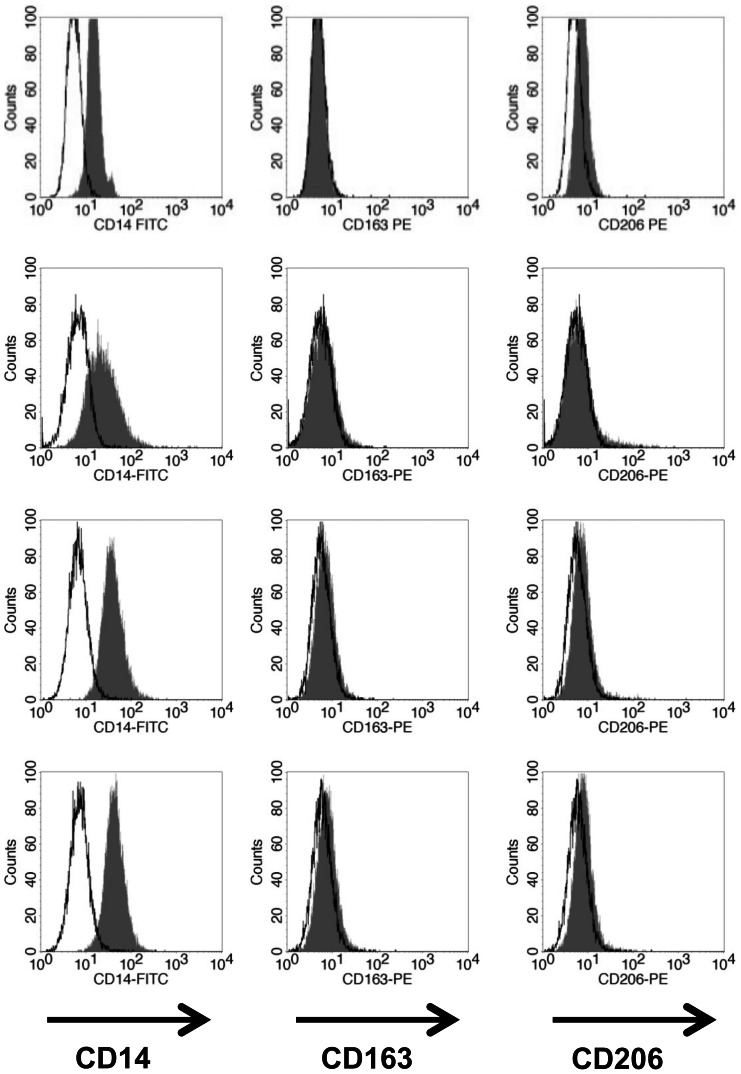
Surface expression of CD14, CD163 and CD206 on untreated THP-1 cells (mock) or after differentiation into macrophage-like cells by stimulation with PMA alone (PMA) or in combination with 50 ng ml^−1^ M-CSF (M+PMA) or GM-CSF (GM+PMA). All experiments were performed in triplicates (n = 3). Histogram plots show surface marker staining as hatched areas and isotype controls as black lines.

Human macrophages were generated from primary human monocytes following positive or negative immunomagnetic selection and differentiated for 7 days in the presence of either GM-CSF (GM-Mφ) or M-CSF (M-Mφ). Additionally, the human cell line THP-1, which upon PMA-stimulation shows a macrophage-like phenotype [29], was used for comparison. All cells were analysed for expression of the classical surface markers mannose receptor (CD206) and scavenger receptor (CD163) [18] by flow cytometry. GM-Mφ displayed a CD14^+^CD163^−^CD206^high^ phenotype and M-Mφ were CD14^+^CD163^high^CD206^low^ irrespective of the method used for monocyte isolation (Fig. 1A and B). No significant difference in mean fluorescence intensity (MFI) of CD206 on GM-Mφ or CD163 on M-Mφ was observed (Fig. 1C). Expression of CD163 and CD206 was specifically induced upon *ex vivo* differentiation since these markers were not expressed on primary monocytes directly after isolation (Fig. S1). THP-1 macrophage-like cells displayed CD14 surface expression but did not express CD163 or CD206 and these markers could not be induced by additional stimulation with PMA alone or in combination with GM-CSF or M-CSF (Fig. 2).

### Viability of macrophages upon infection with *Lm*

**Figure 3:**
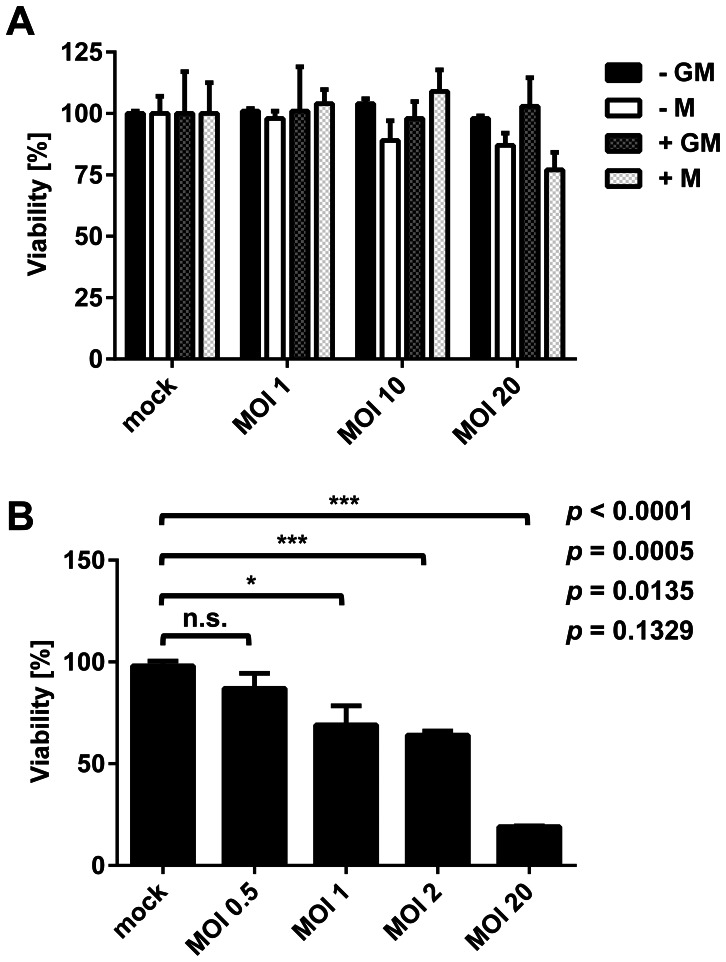
Viability of GM-Mφ and M-Mφ generated from monocytes isolated by positive (+GM or +M) or negative (-GM or -M) selection (A) or PMA-stimulated THP-1 macrophages (B) infected with *Lm* at different multiplicities of infection (MOI). Results from one representative donor (A) or experiment (B) measured in triplicate are shown and similar results were obtained with cells of at least three different donors or in three independent experiments. Statistical analysis was performed by pairwise comparison to the respective uninfected controls using Student's *t*-test.

In contrast to circulating monocytes, tissue macrophages are robust cells and relatively resistant to apoptosis [30]. Viability of all types of macrophages following infection with *Lm* at different MOIs was investigated by MTT conversion. Primary human macrophages tolerated high levels of *Lm* without a significant loss in viability (Fig. 3A). Cells remained completely viable for at least 4 h post infection even at an MOI of 10. A slight decrease in viability to approx. 80% was only observed for M-Mφ at an MOI of 20 (Fig. 3A) but this trend was, however, not statistically significant. By contrast, the viability of PMA-stimulated THP-1 macrophages was dramatically affected even by low doses of *Lm*. As little as 1 bacterium per cell was sufficient to reduce viability of PMA-stimulated THP-1 cells significantly to 69% compared to uninfected controls and at MOI 20 viability was below 20% (Fig. 3B).

Due to the reduced tolerance to *Lm* infection and their lack of markers of macrophage differentiation, PMA-stimulated THP-1 cells were considered inappropriate as model to study infection of human macrophages by *Lm*. Thus, further experiments were carried out with primary human macrophages only.

### Phagocytic activity of GM-Mφ is influenced by the method of monocyte isolation

**Figure 4:**
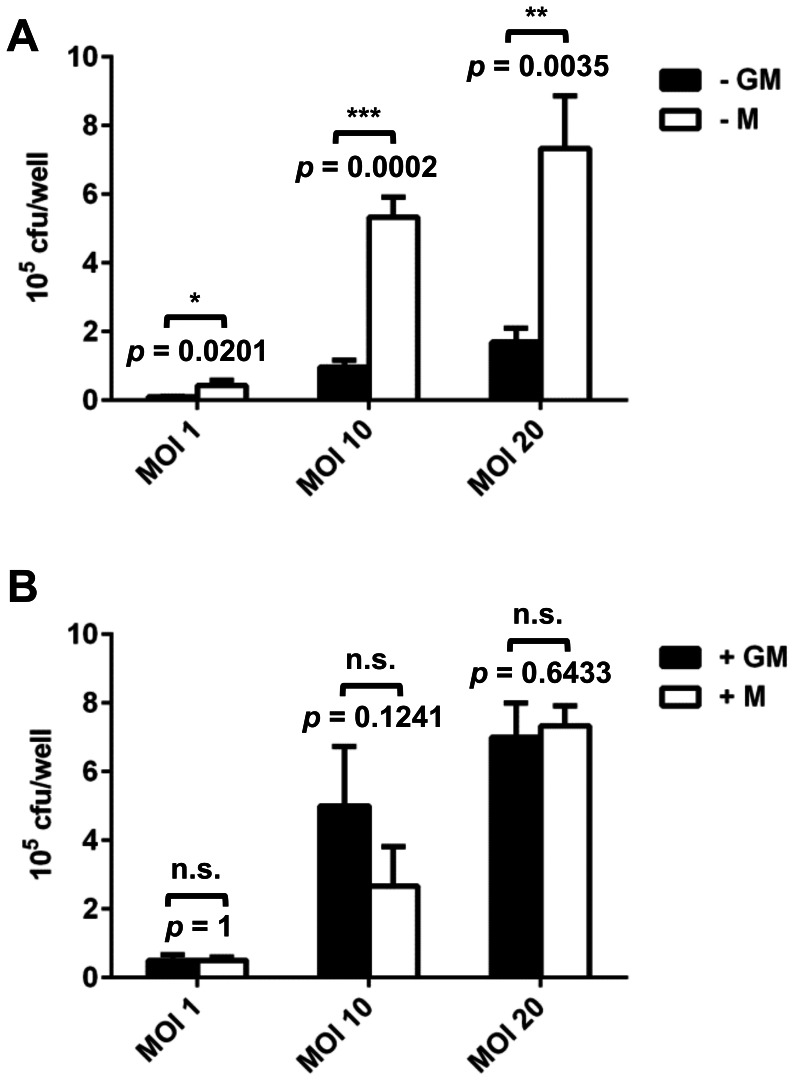
Phagocytosis of *Lm* by GM-Mφ (GM, black bars) or M-Mφ (M, white bars) generated from monocytes isolated by negative (A) or positive (B) selection or THP-1 macrophages (C) at different multiplicities of infection (MOI). Phagocytosis is measured as colony forming units (CFU) per well. Results from one representative donor (A and B) or experiment (C) measured in triplicates are shown and similar results were obtained with cells of at least three different donors or in three independent experiments. Statistical analysis was performed by pairwise comparison of GM-Mφ vs. M-Mφ at the different MOIs using Student's *t*-test (n.s.: not significant).

Besides their anti-inflammatory properties, one of the characteristics of M-Mφ is their high phagocytic activity [14], [16], [31], [32]. In line with these studies, significantly higher phagocytosis of *Lm* by M-Mφ was observed compared to GM-Mφ at all MOIs tested (1, 10, 20) when macrophages were generated from negatively selected monocytes (Fig. 4A). By contrast, no significant difference in the amount of phagocytosed bacteria was observed between M-Mφ and GM-Mφ generated from monocytes isolated by positive selection with both macrophage populations showing similarly high phagocytic activity (Fig. 4B).

**Figure 5:**
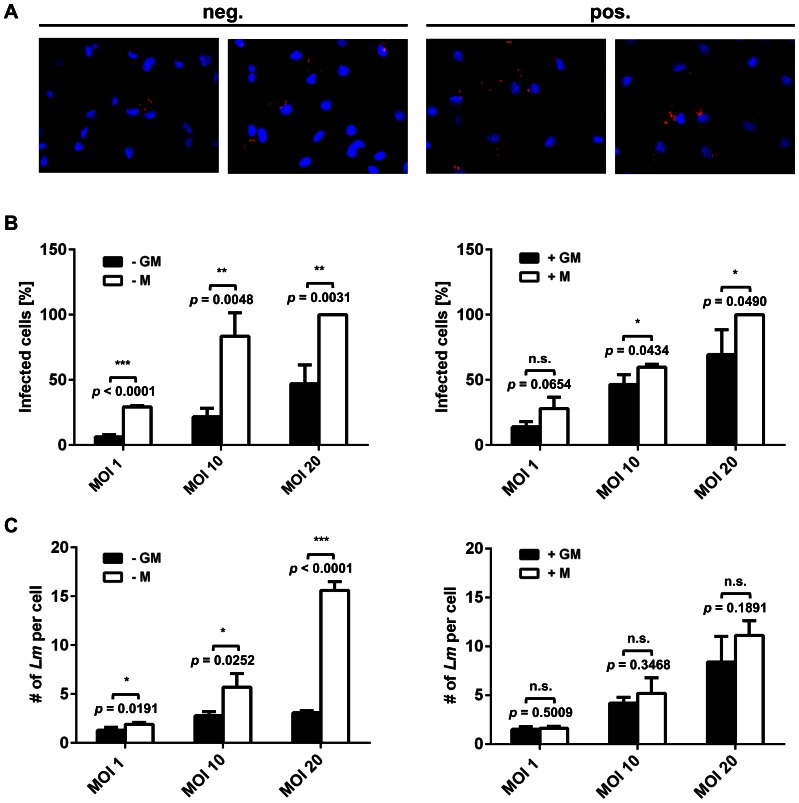
Fluorescence microscopy of GM-Mφ (GM) and M-Mφ (M) generated from monocytes isolated by negative (neg.) or positive (pos.) selection and infected with *Lm* at an MOI of 10 (A). Nuclei of macrophages are stained with DAPI (blue) and *Lm* was stained with a specific antibody (red). Scale bar is 10 µm. Infection of GM-Mφ (-GM or +GM, black bars) and M-Mφ (-M or +M, white bars) with *Lm* was determined at different multiplicities of infection (MOI) and fluorescence microscopy images were analysed either for the percentage of infected cells (B) or the number of bacteria per infected cell (C). For each MOI and macrophage phenotype, infected cells and *Lm* per infected cell were counted in random microscopic fields of view of three donors. For each donor, three independent fields of view with at least 100 cells were analysed. Statistical analysis was performed on the means of different donors by pairwise comparison of GM-Mφ vs. M-Mφ at the different MOIs using Student's *t*-test (n.s.: not significant).

In order to investigate *Lm* infection of GM-Mφ and M-Mφ on a single cell level, infected cells were analysed by fluorescent microscopy (Fig. 5A). Quantitative image analysis confirmed that M-Mφ phagocytosed significantly more *Lm* at all MOIs than GM-Mφ, when macrophages were derived from monocytes isolated by negative selection (Fig 5B and C, left panels). Again, high phagocytosis of both cell types was observed when monocytes were isolated by positive selection (Fig. 5B and C, right panels). At higher MOIs still a minor yet statistically significant difference in phagocytosis of *Lm* by M- and GM-Mφ could be detected when looking at the number of infected cells (Figure 5B, left panel). However, no difference in the amount of intracellular bacteria could be observed between the two cell types (Figure 5C, left panel). More importantly, positive selection of monocytes increased the number of infected cells of GM-Mφ at all MOIs tested.

In all cases infection with higher MOIs resulted in higher number of infected macrophages as well as higher numbers of *Lm* per infected macrophage (Fig. 5B and C). Moreover, even at lower MOIs cells infected by only one bacterium were rarely detected and macrophages with multiple intracellular bacteria were by far more frequent than would be expected by chance (Fig. 5A).

### Phagocytosis of latex beads and other microorganisms

**Figure 6:**
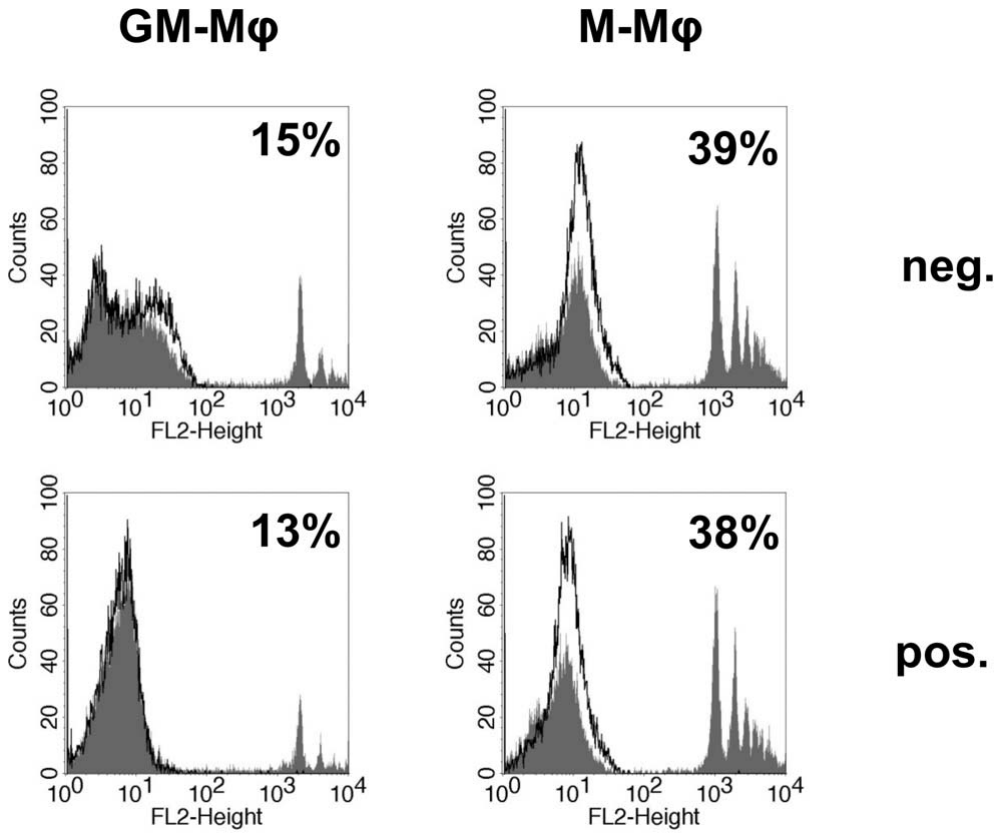
Phagocytosis of fluorescent latex beads by GM-Mφ and M-Mφ generated from monocytes isolated by negative (neg.) or positive (pos.) selection as measured by flow cytometry. Histogram plots show macrophages incubated with latex beads (hatched area) and cells alone (black lines) as control. Percentage of macrophages which are positve for latex beads are indicated in each histogram plot. Results from one representative donor are shown and similar results were obtained with cells of at least three different donors.

Our data strongly suggest that phagocytosis of *Lm* by GM-Mφ is affected by the method used to isolate monocytes. In order to investigate if this holds true for non-specific phagocytosis, experiments were performed using fluorescence latex beads. Again, M-Mφ showed higher phagocytosis than GM-Mφ and this effect was observed irrespective of the method used to isolate monocytes (Fig. 6).

**Figure 7:**
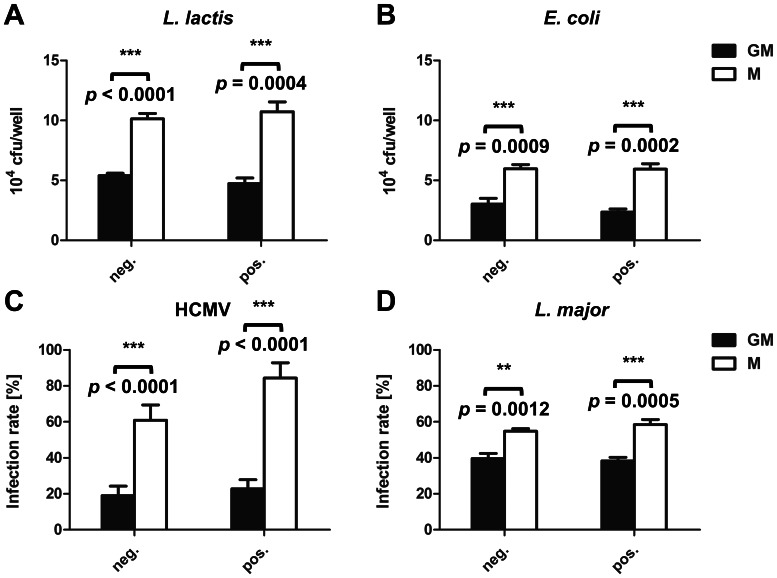
Phagocytosis of *Lactococcus lactis* (A), *Escherichia coli* (B), *Leishmania major* (C) and HCMV (D) by GM-Mφ (GM, black bars) and M-Mφ (M, white bars) generated from monocytes isolated by negative (neg.) or positive (pos.) selection. Cells were infected with all microorganisms at an MOI of 1. Phagocytosis was determined as described in Materials and Methods expressed as CFU/well or infection rate (%). Results from one representative donor measured in triplicate are shown and similar results were obtained with cells of at least three different donors. Statistical analysis was performed by pairwise comparison of GM-Mφ vs. M-Mφ using Student's *t*-test.

Since phagocytosis of latex beads by GM-Mφ was not increased in cells derived from positively isolated monocytes, we next sought to investigate if the method of isolation has an impact on phagocytosis of other microorganisms. To this end, assays were performed using a variety of microorganisms including Gram-negative and -positive non-invasive bacteria (*E. coli* and *L. lactis*), a protozoan parasite (*L. major*) and a virus (human cytomegalovirus; HCMV). As observed for *Lm* and latex beads, M-Mφ showed higher levels of intracellular bacteria, parasites or susceptibility to viral infection than GM-Mφ (Fig. 7). Interestingly, for none of these microorganisms positive selection of monocytes enhanced infection rates by GM-Mφ (Fig. 7).

### Effects of CD14 microbeads and antibody on phagocytosis of *Lm* by GM-Mφ

**Figure 8:**
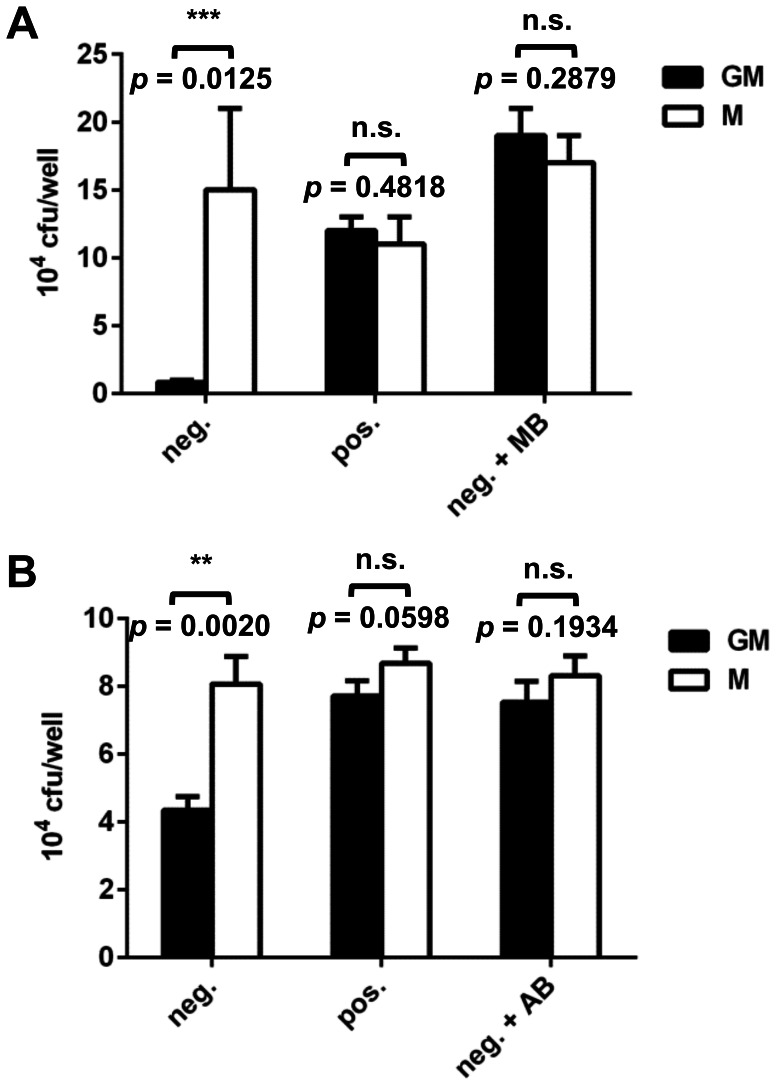
Phagocytosis of *Lm* by GM-Mφ (GM, black bars) and M-Mφ (M, white bars) generated from monocytes isolated by negative (neg.) or positive (pos.) selection as well as negatively isolated macrophages, which were additionally incubated with the CD14 microbeads (neg.+MB; A) or the α-CD14 antibody (neg. +AB; B). Cells were infected with *Lm* at an MOI of 1. Results from one representative donor measured in triplicate are shown and similar results were obtained with cells of at least three different donors. Statistical analysis was performed by pairwise comparison of GM-Mφ vs. M-Mφ using Student's *t*-test (n.s.: not significant).

These results clearly indicate that positive selection has an impact on the phagocytosis of *Lm* by primary human macrophages. Further experiments were performed to exclude differences in the monocyte populations isolated by positive and negative selection and to assess the impact of CD14 microbeads used for positive selection on phagocytic activity. For this purpose, M-Mφ and GM-Mφ derived from monocytes isolated by negative selection were additionally incubated with the CD14 microbeads used for positive selection and then infected with *Lm*. Incubation with CD14 microbeads had no effect on the phagocytic activity of M-Mφ. By contrast, the same treatment resulted in increased phagocytosis of *Lm* by GM-Mφ comparable to that observed with GM-Mφ derived from monocytes after positive selection (Fig. 8A). Non-specific phagocytosis of latex beads was not affected by incubation with CD14 microbeads (Fig. S2). Furthermore, similar results were obtained when macrophages were generated in the presence of the same α-CD14 antibody not coupled to magnetic microbeads during the 7 days of differentiation following negative selection (Fig. 8B). However, the presence of α-CD14 antibody during differentiation did not change phagocytosis of *E. coli* or *L. lactis* by GM-Mφ (Fig. S3).

## Discussion

The first cells of the innate system *Lm* encounters upon infection of a host are resident macrophages of the intestinal mucosa [21], [23]. During later stages of infection, after systemic spread via blood and lymph streams, *Lm* is mainly phagocytosed by Kupffer cells, i.e. tissue-specific macrophages of the liver [2], [3]. Thus, macrophages undoubtedly are an important component of the defence against listerial infections.

*Ex vivo* differentiation of primary human blood monocytes represents a good model to study interactions of pathogens with human macrophages [14], [31], [33]. In line with previous studies [14], [15], [18], [33], *ex vivo* differentiated macrophages showed either high surface expression of CD163 (M-Mφ) or CD206 (GM-Mφ) regardless of the method used to isolate monocytes. Treatment with PMA alone or in combination with M- or GM-CSF did not induce expression of these markers on THP-1 cells. This is in agreement with a previous report in which CD206 could not be induced on THP-1 cells by PMA treatment [29].

GM-Mφ are similar in their functional properties with M1 or classically activated macrophages generated *ex vivo* by treatment with tumour necrosis factor α, interferon γ alone or in combination with lipopolysaccharide and thus reflect inflammatory macrophages that mediate defence of the host during the acute phase of an infection [8], [12], [34], [35]. Accordingly, M-Mφ have anti-inflammatory capacity similar to M2 or alternatively activated macrophages, which can also be generated *ex vivo* from monocytes by incubation with IL-4 and/or IL-13 [8], [12], [34], [35], and mimic resident tissue macrophages.

GM- and M-Mφ generated from monocytes isolated by either positive or negative selection were analysed for phagocytosis and compared to PMA-stimulated THP-1 macrophage-like cells. All M-Mφ were characterised by a high phagocytic activity towards *Lm* and GM-Mφ derived from negatively selected monocytes showed significantly lower phagocytosis of *Lm* (Fig. 4A and B and Fig. 5).

The results on phagocytosis of M-Mφ and GM-Mφ obtained by classical gentamicin protection assays were confirmed independently by fluorescence microscopy (Fig. 5). Moreover, microscopy revealed that macrophages rarely contained a single bacterium. Instead, in almost all cases infected macrophages harboured multiple bacteria. Since the early time point post infection used for fluorescence microscopy (i.e. 2 h post infection) excludes extensive intracellular bacterial replication, this result indicates that phagocytosis of *Lm* by macrophages is somehow activated upon contact with a first bacterium leading to uptake of further bacteria.

Higher phagocytosis of *Lm* by M-Mφ is in line with previous studies showing higher phagocytic activity of M-Mφ (also termed M2 or alternatively activated macrophages) for apoptotic cells, *Mycobacterium bovis* BCG, HCMV and human immunodeficiency virus 1 [14], [16], [31], [32] as compared to GM-Mφ (also termed M1 or classically activated macrophages). The only exception described so far is the Dengue virus, which binds the mannose receptor CD206. This receptor is expressed at higher levels on GM-Mφ compared to M-Mφ leading to higher infection rates in GM-Mφ [36].

Interestingly, positive selection of monocytes promoted phagocytosis of *Lm* by GM-Mφ (Fig. 4B). However, we did not observe enhanced uptake of latex beads, *E. coli*, *L. lactis*, HCMV and *Leishmania major* by GM-Mφ derived from positively isolated monocytes. This suggests that the effect of positive selection on phagocytosis is specific for *Lm*. Further experiments revealed that incubation with CD14 microbeads or α-CD14 antibody alone was sufficient to induce enhanced uptake of *Lm* (Fig. 8) indicating that ligation of CD14, might trigger the phagocytic response. CD14 is an accessory molecule of different toll-like receptors (TLRs) interacting directly with the ligands of TLR2, 3, 4 and 6 [37]. Probably the best characterised function of CD14 is its role as accessory receptor during TLR4-mediated LPS signalling [38]. However CD14 has also been described also as a pattern recognition receptor in innate immunity for a variety of ligands [39]. It is highly expressed on almost all monocytes and macrophages [40] except for resident tissue macrophages of the intestinal mucosa where it is downregulated to maintain tolerance to the intestinal microbiota [21].

On monocytes and macrophages CD14 is bound to the membrane via a glycosylphosphatidylinositol anchor but lacks intracellular domains [41]. Thus, activation of phagocytosis, as well as other downstream responses, by CD14 ligation probably involves other adaptor molecules. In mice, CD14 was shown to cooperate with TLR2 for efficient clearance of *Lm* infections [42]. Also, CD14 has been shown to interact with complement receptor 3 to mediate phagocytosis of *Borrelia burgdorferi* by murine bone marrow-derived macrophages in a process independent of TLR2 [43]. Further studies will be required to identify the signalling cascade activated in human macrophages upon CD14 ligation and the regulatory mechanisms leading to increased phagocytosis of *Lm*.

Based on our results we propose macrophages differentiated *ex vivo* from primary monocytes isolated by negative selection as the best model to study interaction of *Lm* with human macrophages. The use of primary human cells excludes any species-specific biases of murine macrophages. GM- and M-CSF are important mediators of macrophage differentiation. GM-CSF is abundantly expressed at sites of inflammation resulting in recruitment of circulating monocytes and their differentiation into pro-inflammatory Mφ. By contrast, high levels of M-CSF result in an anti-inflammatory phenotype [8]. Thus the use of *ex vivo* differentiated GM-Mφ or M-Mφ allows the investigation of *Lm* infections in macrophages of different phenotypes. With respect to *Lm* infection, M-Mφ might be of particular relevance since they share phenotypic and functional properties of resident macrophages in the intestinal mucosa [44], which is the primary site of *Lm* infection.

In summary, we could show that GM-Mφ and M-Mφ display different phagocytic activity towards *Lm*. Moreover, our data suggests that ligation of CD14 on GM-Mφ enhances phagocytosis of *Lm* but does not affect high phagocytic activity of M-Mφ. The enhancing effect of CD14 ligation appeared to be specific for *Lm* since internalisation of other pathogens and latex beads was not enhanced.

## Supporting Information

Figure S1 **Surface expression of CD14, CD163 and CD206 on monocytes isolated from human buffy coats by positive (pos.) or negative selection alone (neg.) or in combination with CD14 microbeads (neg. +MB) as analysed by flow cytometry.** Histogram plots show surface marker staining as hatched areas and isotype controls as black lines. Results from one representative donor are shown and similar results were obtained with cells of at least three different donors.
(TIF)

Figure S2 **Phagocytosis of fluorescent latex beads by GM-Mφ and M-Mφ generated from monocytes isolated by negative selection and additionally incubated with CD14 microbeads (neg. +MB) as measured by flow cytometry.** Histogram plots show macrophages incubated with latex beads (hatched area) and cells alone (black lines) as control. Percentage of macrophages which are positve for latex beads are indicated in each histogram plot. Results from one representative donor are shown and similar results were obtained with cells of at least three different donors.
(TIF)

Figure S3 **Phagocytosis of** ***Lactococcus lactis*** **(A) and** ***Escherichia coli*** **(B) by GM-Mφ (GM, black bars) and M-Mφ (M, white bars) generated from monocytes isolated by negative (neg.) or positive (pos.) selection as well as negatively isolated macrophages, which were additionally incubated with α-CD14 antibody (neg. +AB).** Cells were infected with bacteria at an MOI of 1. Results from one representative donor measured in triplicate are shown and similar results were obtained with cells of at least three different donors. Statistical analysis was performed by pairwise comparison of GM-Mφ vs. M-Mφ using Student's *t*-test.
(TIF)
