# Supplementary figures and images for: CD14-Dependent Monocyte Isolation Enhances Phagocytosis of Listeria monocytogenes by Proinflammatory, GM-CSF-Derived Macrophages
Source: PLoS One. 2013 Jun 11;8(6):e66898. doi: 10.1371/journal.pone.0066898 (PMC3679097; doi:10.1371/journal.pone.0066898)

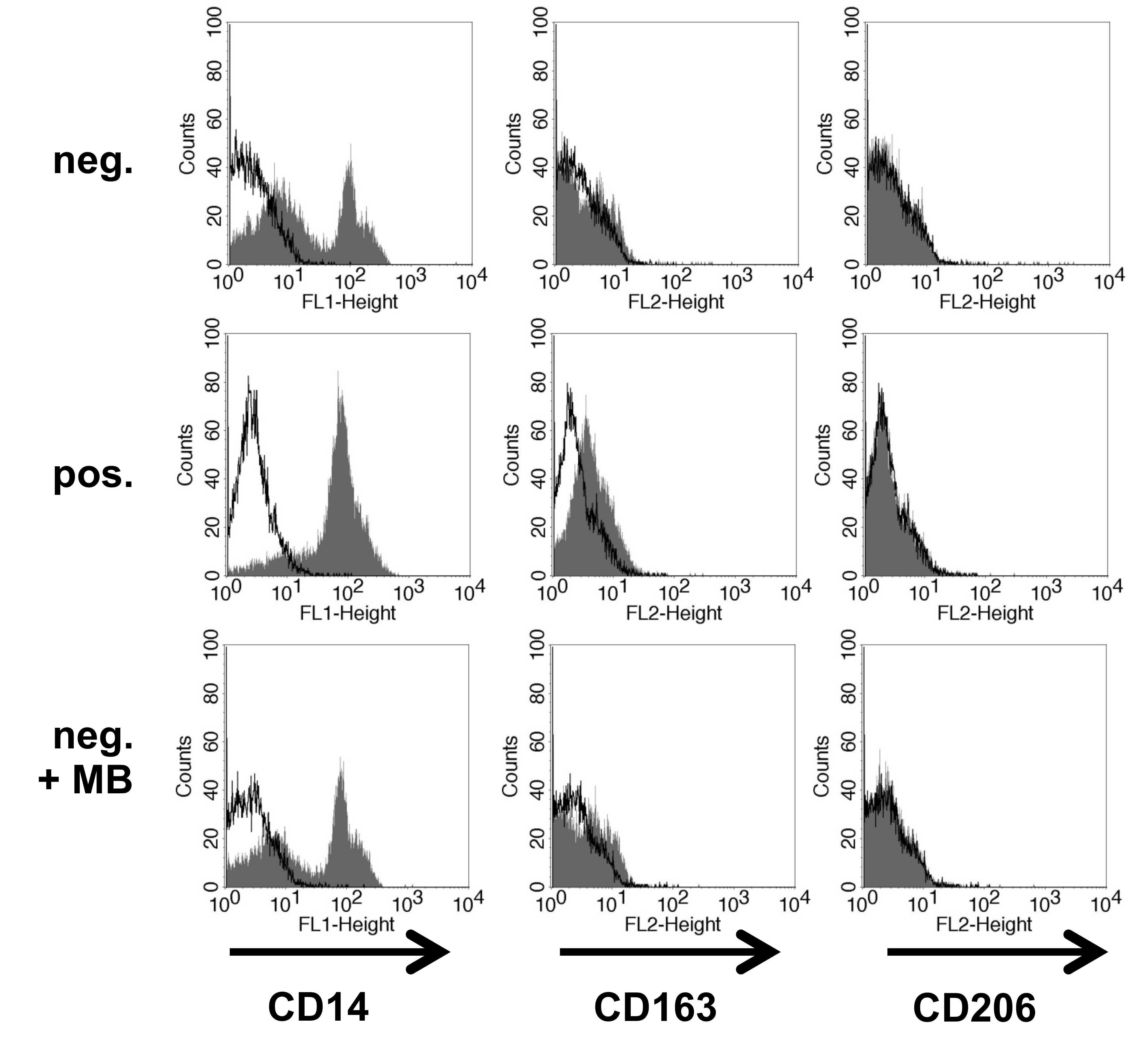

Supplement: Figure S1 — Surface expression of CD14, CD163 and CD206 on monocytes isolated from human buffy coats by positive (pos.) or negative selection alone (neg.) or in combination with CD14 microbeads (neg. +MB) as analysed by flow cytometry. Histogram plots show surface marker staining as hatched areas and isotype controls as black lines. Results from one representative donor are shown and similar results were obtained with cells of at least three different donors. (TIF) [file pone.0066898.s001.tif]

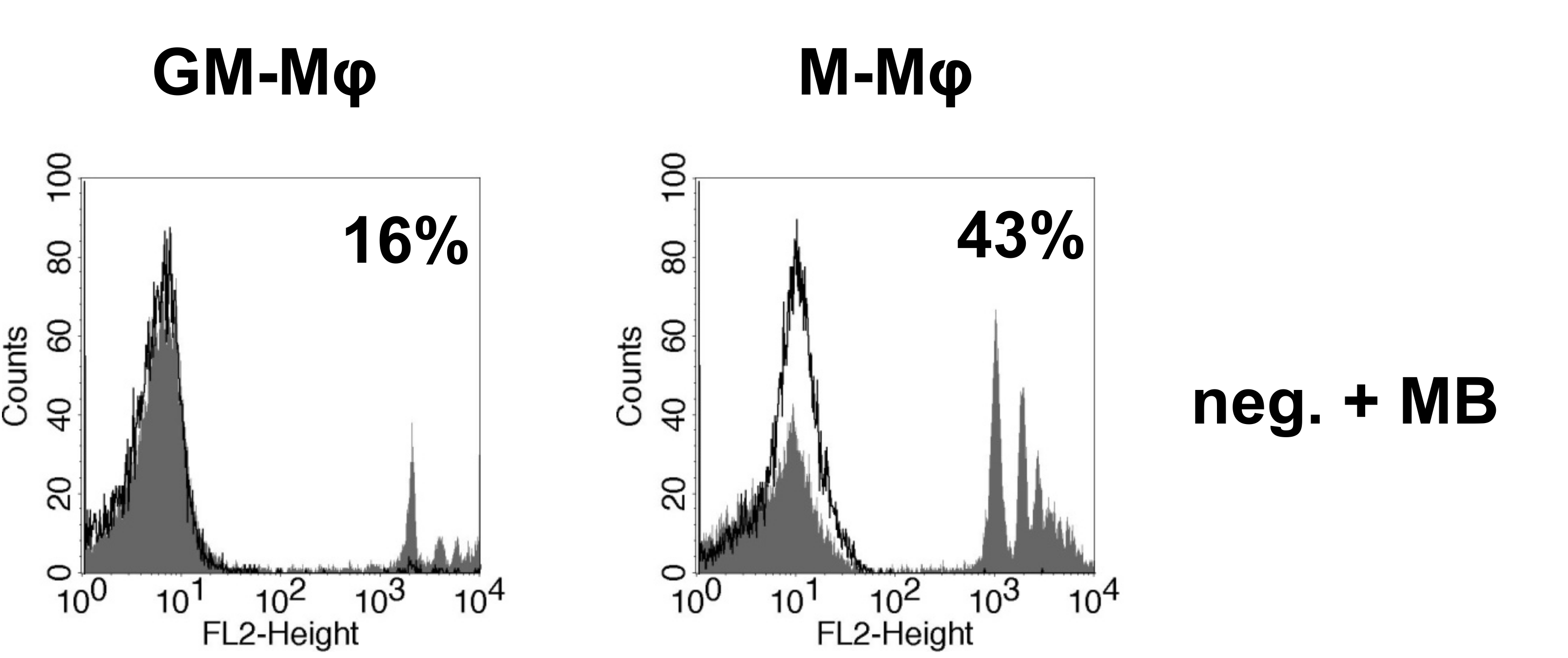

Supplement: Figure S2 — Phagocytosis of fluorescent latex beads by GM-Mφ and M-Mφ generated from monocytes isolated by negative selection and additionally incubated with CD14 microbeads (neg. +MB) as measured by flow cytometry. Histogram plots show macrophages incubated with latex beads (hatched area) and cells alone (black lines) as control. Percentage of macrophages which are positve for latex beads are indicated in each histogram plot. Results from one representative donor are shown and similar results were obtained with cells of at least three different donors. (TIF) [file pone.0066898.s002.tif]

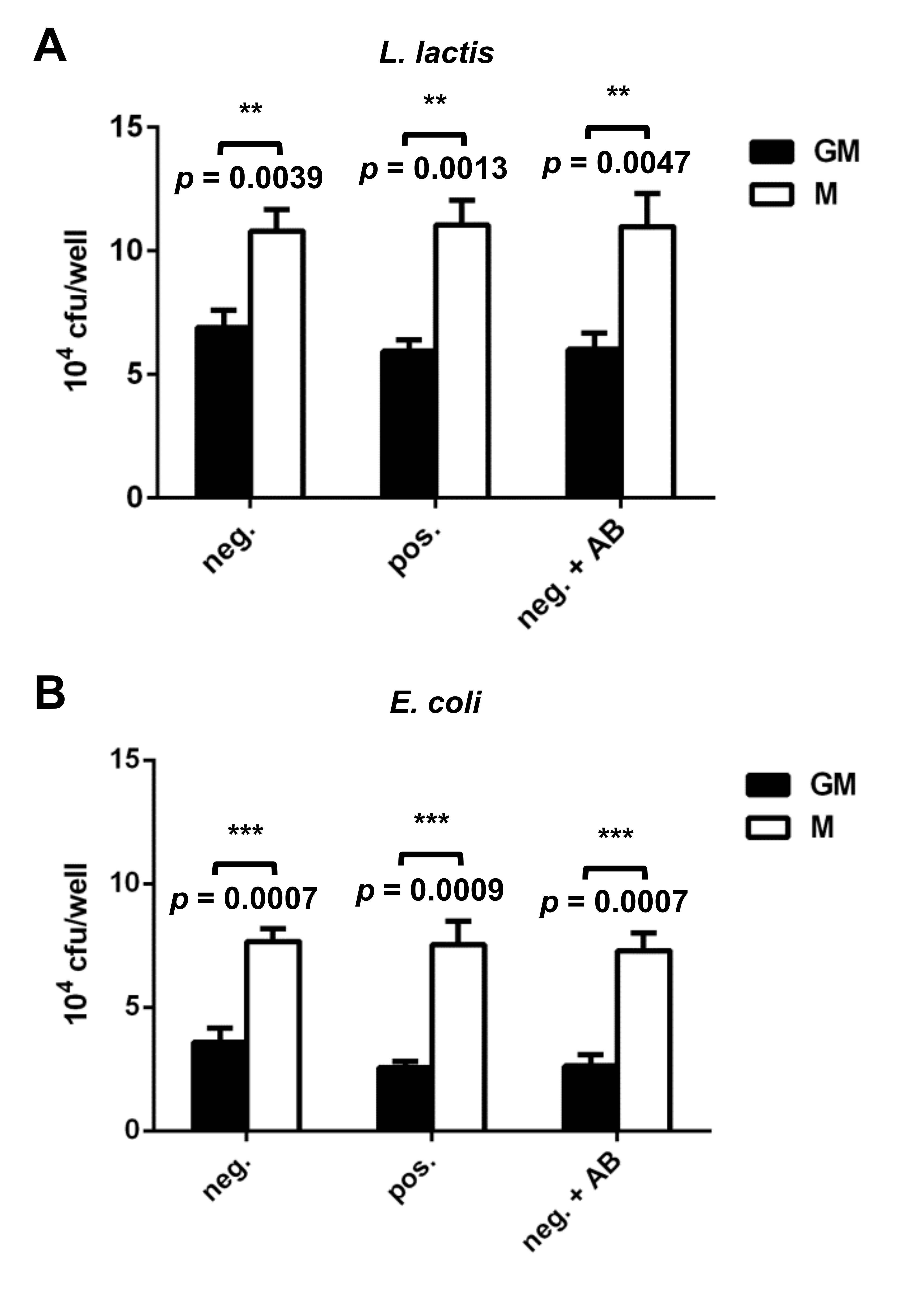

Supplement: Figure S3 — Phagocytosis of Lactococcus lactis (A) and Escherichia coli (B) by GM-Mφ (GM, black bars) and M-Mφ (M, white bars) generated from monocytes isolated by negative (neg.) or positive (pos.) selection as well as negatively isolated macrophages, which were additionally incubated with α-CD14 antibody (neg. +AB). Cells were infected with bacteria at an MOI of 1. Results from one representative donor measured in triplicate are shown and similar results were obtained with cells of at least three different donors. Statistical analysis was performed by pairwise comparison of GM-Mφ vs. M-Mφ using Student's t-test. (TIF) [file pone.0066898.s003.tif]
